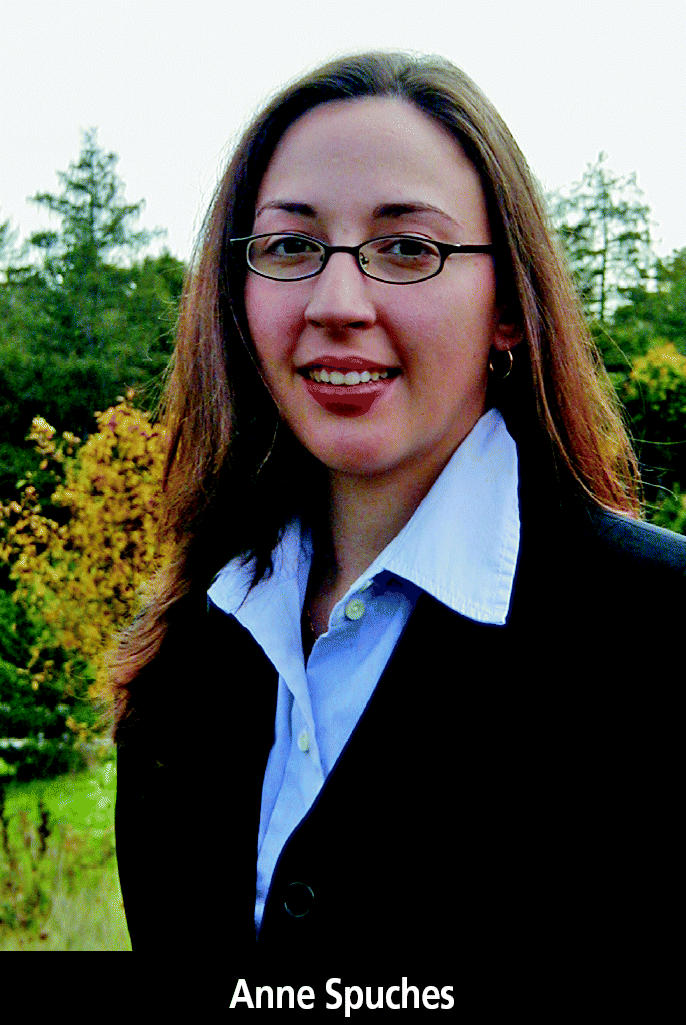# Anne Spuches Receives 2004 Karen Wetterhahn Memorial Award

**Published:** 2004-12

The Superfund Basic Research Program (SBRP) is pleased to announce that Anne Spuches of Dartmouth College is the recipient of the seventh annual Karen Wetterhahn Memorial Award. The award was presented to Spuches on 4 November 2004 at the SBRP Annual Meeting at the University of Washington in Seattle.

The SBRP presents this annual award to an outstanding scholar to pay tribute to the life and scientific accomplishments of Karen E. Wetterhahn, former director of the SBRP at Dartmouth College. Wetterhahn died in 1997 as the result of an accidental exposure to dimethylmercury. An acknowledged international expert on the effects of heavy metals on biological systems, Wetterhahn was a leader in conducting research on how metals initiate cancer and other metal-induced human diseases at the molecular level. She fostered links between biology, chemistry, environmental studies, engineering, and medical science, insisting that “the life sciences are interdisciplinary.”

Spuches, who earned her Ph.D. in chemistry at Yale University, is in her second year as a postdoctoral fellow at Dartmouth College. Advised by professor Dean E. Wilcox, she is participating in interdisciplinary studies addressing the environmental and human health effects of arsenic. The toxicity of arsenic at low chronic exposure, primarily through arsenite in drinking water, poses a significant health risk for people around the world. Specifically, Spuches is using isothermal titration calorimetry to quantify the interaction of arsenite and monomethylarsenite with various thiols. This information is fundamental to mapping the distribution and chemistry of arsenic in the cell, and may also help in the design of more effective chelating agents for the treatment of arsenic poisoning.

The NIEHS congratulates Spuches on her research accomplishments and wishes her continued success in her scientific career.

## Figures and Tables

**Figure:**